# Hemodynamic monitoring in sepsis and septic shock in pediatric patients: a multicenter survey

**DOI:** 10.62675/2965-2774.20260262

**Published:** 2026-05-05

**Authors:** Carolina Barone Quintella Cury, Daniela Carla de Souza, Orlei Ribeiro de Araujo, Cristian Tedesco Tonial, José Colleti, Arnaldo Prata-Barbosa, Vanessa Soares Lanziotti

**Affiliations:** 1 Universidade Federal do Rio de Janeiro Instituto de Puericultura e Pediatria Martagão Gesteira Postgraduate Program in Child and Maternal Health Rio de Janeiro RJ Brazil Postgraduate Program in Child and Maternal Health - Instituto de Puericultura e Pediatria Martagão Gesteira, Universidade Federal do Rio de Janeiro - Rio de Janeiro (RJ), Brazil.; 2 Univesidade de São Paulo Hospital Universitário São Paulo SP Brazil Hospital Universitário, Univesidade de São Paulo - São Paulo (SP), Brazil.; 3 Universidade Federal de São Paulo Instituto de Oncologia Pediátrica Grupo de Apoio ao Adolescente e à Criança com Câncer São Paulo SP Brazil Grupo de Apoio ao Adolescente e à Criança com Câncer, Instituto de Oncologia Pediátrica, Universidade Federal de São Paulo - São Paulo (SP), Brazil.; 4 Universidade Federal do Rio Grande do Sul Hospital de Clínicas de Porto Alegre Porto Alegre RS Brazil Hospital de Clínicas de Porto Alegre, Universidade Federal do Rio Grande do Sul - Porto Alegre (RS), Brazil.; 5 Faculdade de Medicina de Jundiaí São Paulo SP Brazil Faculdade de Medicina de Jundiaí - São Paulo (SP), Brazil.

**Keywords:** Sepsis, Shock, septic, Hemodynamic monitoring, Survey, Child, Intensive care units, pediatric, Surveys and Questionnaires

## Abstract

**Objective:**

To evaluate the current practices of hemodynamic monitoring in sepsis and septic shock in Brazilian pediatric intensive care units.

**Methods:**

A multicenter, cross-sectional study using an electronic survey.

**Results:**

An electronic survey was responded to in three rounds (1 week each), and responses from 77 pediatric intensive care units in 26 states of Brazil were collected. Point-of-care cardiac ultrasound was the method of choice for fluid responsiveness evaluation in septic children in 61% pediatric intensive care units; 78% pediatric intensive care units had enough arterial line monitors available to every bed, but only 57% use invasive arterial blood pressure monitoring for every patient diagnosed with septic shock; 22% use invasive arterial blood pressure monitoring just for refractory shock patients. To guide initiation/titration of inotropes and vasoactive agents, 70% pediatric intensive care units said their decision is based on clinical assessment; 20% use point-of-care cardiac ultrasound to aid vasoactive agent choice; 88% pediatric intensive care units use Lactate trend, and 35% measure central/mixed venous saturation in patients with septic shock.

**Conclusion:**

Differences between international guidelines and practice in Brazilian pediatric intensive care units regarding hemodynamic monitoring in pediatric sepsis were identified. These differences may arise for several reasons; recognizing them is crucial to making appropriate changes and adjusting valuable guidelines to make them more feasible for low- and middle-income countries.

## INTRODUCTION

Sepsis and septic shock remain a serious public health issue worldwide, being a major cause of morbidity and mortality in pediatric intensive care units (ICUs), even in high-income countries.^(1)^ The mortality rate in pediatric sepsis can reach up to 40% in low and middle-income countries,^(2)^ and it varies significantly according to geographic region, with mortality rates being approximately 4.4 times higher in low- and middle-income countries compared to high-income countries.^(2-4)^ In Brazil, those numbers can reach 30% in some regions,^(5)^ pointing out the need for localized studies, as current recommendations are mostly based on data from high-income countries, and may not be applicable in the particular contexts of low- and middle-income countries^(2)^ due to disparities in the available resources in pediatric ICUs and the technical training of health care professionals.^(6-8)^

Sepsis is currently defined as an organ dysfunction caused by a dysregulated host response to an infectious agent.^(3,9,10)^ Hemodynamic decompensation in sepsis involves complex physiological interactions that encompass vascular tone, hypovolemia, and myocardial dysfunction,^(11)^ and it is known that physical examination alone is often not sensitive enough to differentiate these subtle changes in the disease's pathophysiology.^(12,13)^ In Latin American countries, studies reveal that a large proportion of children admitted to pediatric ICUs receive inadequate initial volume resuscitation and approximately 35% of them die in the early stages of the disease (less than 72 hours) due to refractory septic shock, with that being the most common cause of death in pediatric sepsis.^(6,14)^ The 2020 Surviving Sepsis Campaign (SSC 2020) guidelines recommend using advanced hemodynamic variables, such as cardiac output, systemic vascular resistance, and central venous oxygen saturation, alongside clinical examination, to guide resuscitation. Also, bedside echocardiography and invasive arterial pressure monitoring are emphasized as valuable tools to improve diagnostic accuracy and optimize therapeutic interventions.^(1)^

However, hemodynamic monitoring, essential for cardiovascular assessment and stabilization in shock, still faces significant obstacles in limited resources regions, which highlights the importance of identifying discrepancies in clinical practices between different regions^(8,15,16)^ and developing guidelines adapted to different realities, incorporating effective measures to improve adherence to best practices and, consequently, clinical outcomes in pediatric patients with sepsis and septic shock.^(1,15,17-19)^

This study aimed to evaluate the current practices of hemodynamic monitoring in sepsis and septic shock in Brazilian pediatric ICU.^(1)^ We also performed stratified comparisons between public and private/philanthropic hospitals to give an additional context for the availability and use of hemodynamic monitoring in different resource settings.

## METHODS

This was a cross-sectional observational study, structured as an electronic survey, collected in three 1-week periods in 2024. The present study was strictly observational and centered on institutional processes rather than patients’ information, and it did not interfere with clinical management or the clinical decision-making process. The Research Ethics Committee of *Instituto de Puericultura e Pediatria Martagão Gesteira* of the *Universidade Federal do Rio de Janeiro*, approved the study (nº 6.212.523, August 2023). As no intervention was performed, Informed Consent was waived.

### Questionnaire development

The questionnaire was composed of objective and closed questions and reviewed by the group of authors in this study, all of whom are board-certified pediatric ICU specialists, through a qualitative expert review^(20)^ to ensure inclusion of basic characteristics of each responding unit and alignment with SSC 2020 recommendations.^(1)^

For this survey, the SSC 2020^(1)^ recommendations were translated into three-level responses as to assess if the practices of respondents were concordant, partially concordant or non-concordant with benchmark domains such as: initial fluid strategy, timing for initiation of vasoactive agents, hemodynamic assessment after interventions, advanced hemodynamic monitoring (such as invasive blood pressure [IBP] and point-of-are cardiac ultrasound [POCUS]) to guide clinical decisions, lactate measurement/trending and availability/use of an institutional protocol.

The questionnaire underwent peer review and was tested in a pilot study conducted across ten pediatric ICUs. Feedback on item comprehension and the response process was requested from participants in the pilot study, and an evaluation of internal consistency was conducted post hoc. Some demographic questions that did not provide relevant information were excluded, and minor wording adjustments were made for greater clarity. Therefore, as there were no major changes in the questionnaires, the ten pilot study responses were used in the final analysis.

The questionnaire was developed and distributed using REDCap®, an online platform for research data collection. We recruited a multicenter convenience sample within the Brazilian Research Network in Pediatric Intensive Care (BRnet-PIC) WhatsApp® group, a collaborative network between pediatric intensive care units. All network pediatric ICUs were invited to complete an institutional survey (one response per unit, preferably answered by the coordinator, but could be answered by a senior staff member) during those three 1-week periods via REDCap®. There was no prior training for the respondents before the survey was distributed, no probability sampling or weighting was applied, and we did not target national representativeness beyond the network. Only fully completed questionnaires were considered, and duplicate questionnaires were treated as a single response.

### Statistical analysis

The statistics were primarily descriptive, using frequencies and percentages. We conducted descriptive statistics and pre-specified univariable comparisons. For comparisons of some frequencies, the simple Chi-squared test and the multiple proportions test were used (with Yates’ correction when indicated).

In addition to the primary descriptive analyses, we conducted exploratory models to contextualize potential associations; findings should be interpreted with caution.

We performed exploratory logistic regression analyses to assess associations between institutional covariates and selected binary practice outcomes. Candidate covariates were public sector hospital (public *versus* private/philanthropic), any accreditation (yes/no), university affiliation (yes/no), non-cardiac clinical + surgical service profile (yes/no), and all shifts staffed exclusively by pediatric intensivists (yes/no). Practice outcomes were not testing fluid responsiveness prior to fluid bolus; not measuring central venous oxygen saturation (ScvO_2_) in septic shock; performing serial lactate monitoring; having a septic shock protocol followed by all professionals; availability of other advanced hemodynamic monitoring methods; initiation/titration of vasoactive agents mostly guided by hemodynamic monitoring; and choice of the initial vasoactive agent based on echocardiography.

We first fitted bivariate logistic models; covariates with p < 0.10 were entered into multivariable models (limited to preserve a reasonable events-per-variable ratio). Results are reported as odds ratios (OR) with 95% confidence intervals (95%CI).

Given the sample size and potential collinearity between institutional factors, these analyses are hypothesis-generating, and we did not adjust for multiplicity. Therefore, transparent descriptive estimates consistent with the study scope were prioritized.

We also compared responses between public and private/philanthropic hospitals to provide additional context on sepsis management across different resource settings.

Demographic data outside the study's sample were retrieved from the Ministry of Health's website (DATASUS)^(21)^ and the EpiMed Database®/*Associação de Medicina Intensiva Brasileira* (AMIB) census^(22)^ and used for external validation.

A single investigator performed data entry, and data consistency was assessed using a 10% random sample verification procedure. Data were screened in detail for missing information, implausible values, and outliers. Analyses were performed using R software (R Foundation for Statistical Computing, Vienna, Austria). A p value < 0.05 was adopted as statistical significance.

In full disclosure, ChatGPT was used to assist in translating the article from Portuguese to English, considering that the authors are not native English speakers, and the authors take full responsibility for the content.

## RESULTS

The characteristics and profiles of the pediatric intensive care units participating in the study are present in table 1 and comparisons by hospital type (Public/SUS and private/philanthropic) are shown in table 2.

**Table 1:** Characteristics and profiles of the pediatric intensive care units participating in the study

Category		Pediatric ICUs n (%)
Hospital type		
	Public - direct administration	17 (22.0)
	Public - outsourced administration	16 (20.8)
	Private without SUS	32 (41.6)
	Private with SUS	3 (3.9)
	Philanthropy with SUS	7 (9.1)
	Philanthropy without SUS	2 (2.6)
Exclusively a pediatric hospital		
	Yes	19 (24.7)
With the emergency department		
	Yes	59 (76.6)
Number of pediatric beds		
	Less than 20	16 (20.8)
	21 to 50	33 (42.8)
	Over 51	28 (36.3)
Pediatric ICU occupancy rate		
	Less than 50%	2 (2.6)
	51 to 65%	10 (12.9)
	66 to 90%	46 (59.7)
	Above 91%	19 (24.6)
Pediatric/neonatal care		
	In the same unit	19 (24.6)
	In separate units	8 (10.4)
	Only pediatric	50 (64.9)
Pediatric ICU profile		
	Clinical and surgical	37 (47.4)
	Specialized	7 (9.0)
	Clinical only	4 (5.2)
	Clinical and surgical (non-cardiac and cardiac)	28 (36.3)
	Cardiac surgery only	1 (1.3)
University connection/pediatric ICU fellowship		
	Connection with university + pediatric ICU fellowship	23 (29.8)
	No connection with the university, but has a pediatric ICU fellowship	20 (25.9)
	No connection and no pediatric ICU fellowship	27 (35.0)
	Connection to the university, but no pediatric ICU fellowship	7 (9.0)
Certification		
	International certification	25 (32.4)
	National certification	24 (31.2)
	No certification	28 (36.3)

ICU - intensive care unit; SUS - *Sistema Único de Saúde*.

**Table 2:** Characteristics and profiles of the pediatric intensive care units participating in the study in relation to the type of hospital

		All pediatric ICUs n (%)	Private/philanthropic n (%)	Public (SUS) n (%)	p valor
Are hospitals accredited by any organization (e.g., ONA, JCI)?					
	International	25 (32.5)	21 (47.7)	4 (12.2)	< 0.001
	National	24 (31.2)	18 (41)	16 (8.1)	0.033
	Not accredited	28 (36.3)	5 (11.3)	23 (69.7)	< 0.001
Number of pediatric ICU beds					
	5 - 10 beds	49 (63.7)	23 (52.3)	26 (78.8)	0.017
	11 - 20 beds	17 (22.1)	13 (29.5)	4 (12.1)	0.07
	> 20 beds	11 (14.3)	8 (18.2)	3 (9.1)	0.26
Occupation rate					
	< 50%	2 (2.6)	2 (4.5)	0	0.21
	51 - 65%	10 (13)	9 (20.4)	1 (3)	0.024
	66 - 90%	46 (59.7)	26 (59)	20 (60.6)	0.89
	> 91%:	19 (24.7)	7 (15.9)	12 (36.3)	0.039
Unit profile					
	Clinic and surgery (non-cardiac)	37 (48)	18 (40.9)	19 (57.5)	0.14
	Clinic and Surgery (both cardiac and non-cardiac)	28 (36.4)	22 (50)	6 (18.2)	0.004
	Only clinic	4 (5.2)	2 (4.5)	2 (6)	0.76
	Another type of specialized ICU	8 (10.4)	2 (4.5)	6 (18.1)	0.052

ICU - intensive care unit; SUS - *Sistema Único de Saúde*; ONA - National Accreditation Organization; JCI - Joint Commission International.

### Overview

Descriptive results for all pediatric ICUs are provided in the "All pediatric ICUs" (n = 77) column of tables 2 - 6. Between-group comparisons were limited to the pre-specified contrast between public Unified Health System (SUS - *Sistema Único de Saúde*) and private/philanthropic hospitals for selected outcomes. We did not perform statistical tests within subcategories of the same multi-option variable.

### Response rate and sample

**Figure 1:**
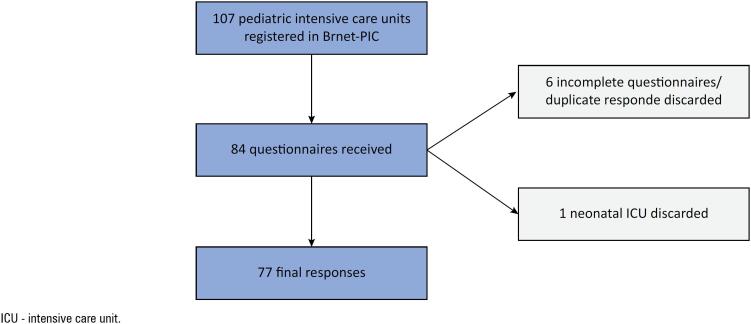
Flowchart representing the selection process of the pediatric intensive care units participating in the research.

Among the 107 pediatric ICUs currently part of BRnet-PIC, 84 units responded to the questionnaires. After excluding duplicate/incomplete questionnaires, 77 pediatric ICUs, each representing a distinct hospital (72% of the total pediatric ICUs in BRnet-PIC), were included in the analysis. A flowchart of the study is shown in figure 1.

**Figure 2:**
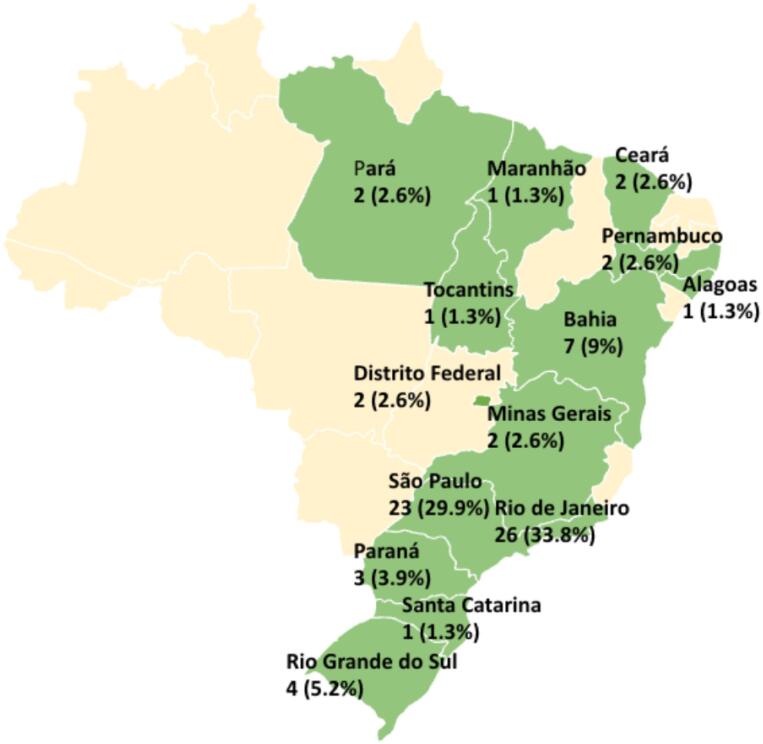
The yellow areas represent the states where there were no responses.

The map in figure 2 shows the geographic distribution of responding pediatric ICUs across Brazil.

The states of São Paulo and Rio de Janeiro accounted for 63.7% of the responses.

### Hospital and pediatric intensive care unit characteristics

The majority of pediatric ICUs surveyed had between five and ten beds (63.6%) and a typical occupancy rate of 66 - 90% (59.7%). Hospitals were exclusively pediatric in 24.7%, and 76.6% had an Emergency Department. Total pediatric bed capacity varied, with 42.8% reporting 21 - 50 pediatric beds. Combined pediatric and neonatal care occurred in 24.6% of units, while 64.9% reported exclusive pediatric care. As for hospital type, 33 of 77 (42.9%) were public (SUS), and 44 of 77 (57.1%) were private/philanthropic. Residency/pediatric ICU Fellowship was present in 55.7% of units. International and national accreditation was reported by 32.4% and 31.2% of hospitals, respectively, while 36.3% were not accredited.

### Staffing, cardiology support, and POCUS availability/training

Table 3 shows the reported availability of pediatric ICU specialists/cardiology support and ultrasound device/trained professionals to perform POCUS, comparing public (SUS) and private/philanthropic hospitals. There were differences in the responses regarding 24-hour cardiologists’ availability, 50% of private/philanthropic hospitals reported having cardiologists available 24 hours a day, and 22.8% reported having cardiologists available only on some days of the week. In public (SUS) hospitals, those numbers were 27.3% and 45.4%, respectively.

**Table 3:** Availability of pediatric intensive care unit specialists/ cardiology support and ultrasound device/trained professionals to perform point-of-care ultrasound

		All pediatric ICUs n (%)	Private/philanthropic n (%)	Public (SUS) n (%)	p valor
Proportion of pediatric intensive care specialists working in pediatric ICUs					
	All the professionals in the unit are specialists	33 (42.9)	18 (40.9)	15 (45.3)	0.69
	Most of the professionals in the unit are specialists (> 50% of the team) AND there's at least one specialist per shift	24 (31.1)	16 (36.3)	8 (24.3)	0.26
	Most of the professionals in the unit are specialists (> 50% of the team), BUT there aren't specialists in every shift	10 (13)	6 (13.6)	4 (12.2)	0.84
	Less than 50% of the professionals in the unit are specialists	10 (13)	4 (9.2)	6 (18.2)	0.24
Availability of an ultrasound device to perform bedside echocardiography					
	There's a device in the pediatric ICU for exclusive use	39 (50.6)	18 (40.9)	21 (63.3)	0.048
	There's a device in the hospital for shared use	34 (44.2)	23 (52.3)	11 (33.4)	0.09
	Not available	4 (5.2)	3 (6.8)	1 (3.3)	0.45
Availability of cardiology service in the hospital					
	Available 24 hours	31 (40.2)	22 (50)	9 (27.3)	0.044
	Available every day during the day	16 (20.8)	10 (22.7)	6 (18.2)	0.62
	Only available on some days of the week	25 (32.5)	10 (22.8)	15 (45.3)	0.035
	Not available	5 (6.5)	2 (4.5)	3 (9.2)	0.42
Proportion of qualified and trained professionals to perform bedside echocardiography					
	There's at least 1 qualified and trained professional on every shift	33 (42.9)	11 (25)	12 (36.3)	0.28
	There are some qualified and trained professionals in the team, but not on every shift	46 (59.7)	28 (63.6)	18 (54.5)	0.42
	There aren't qualified and trained professionals in the team	8 (10.4)	5 (11.4)	3 (9.2)	0.74

ICU - intensive care unit; SUS - *Sistema Único de Saúde*.

Availability of an echocardiography device for exclusive use in the pediatric ICU was reported by 40.9% of private/philanthropic hospitals and 63.6% of public (SUS) hospitals (p = 0.016). 52.3% of public (SUS) hospitals had an ecocardiography device available in the hospital, and 3.3% reported not having devices available, whereas those numbers in private/philanthropic hospitals were 33.4% and 6.8%, respectively (p = 0.57). Regarding training to perform POCUS, the item: "at least one qualified and trained professional in every shift" was more often reported by public (SUS) units (36.3%) than private/philanthropic (25%), p = 0.03.

**Figure 3:**
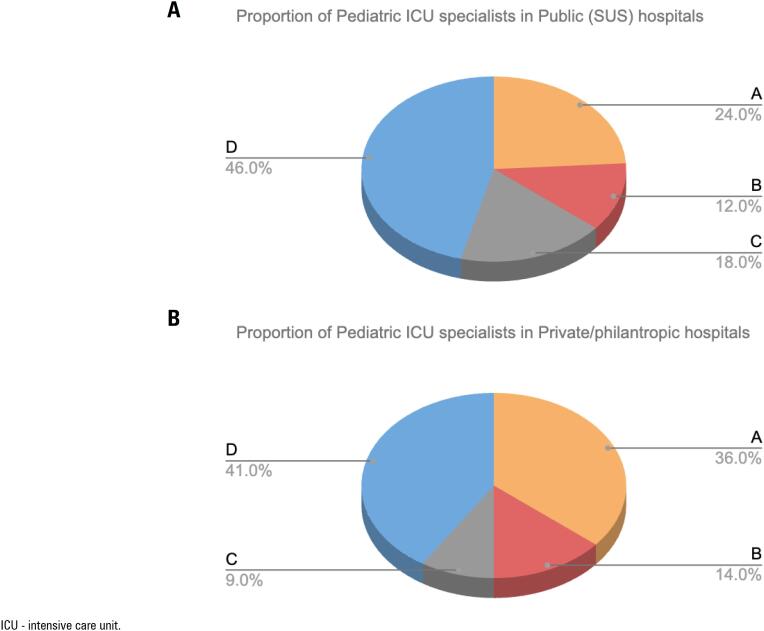
Proportion of pediatric intensive care unit specialists comparing public (Unified Health System) and private/philanthropic hospitals.

For visual representation, figure 3 shows the proportion of pediatric ICU specialists comparing public (SUS) and private/philanthropic hospitals.

(A) Most professionals are pediatric intensive care unit specialists (> 50% of the team) and have at least one pediatric intensive care unit specialist per shift; (B) Most professionals are pediatric intensive care unit specialists (> 50% of the team), but do not have pediatric intensive care unit specialists on all shifts; (C) Less than 50% of the professionals in the unit are pediatric intensive care unit specialists; (D) All professionals are pediatric intensive care unit specialists. The proportions are similar by the Chi-squared for multiple proportions – p = 0.6.

### Hemodynamic monitoring and sepsis management protocol

A sepsis protocol followed by all professionals was reported by 61.3% of private/philanthropic units and by 33.3% of public (SUS) units. In both groups, most units (94.4% of private/philanthropic and 79.2% of public) that reported having a protocol considered it aligned with local practices.

As for IBP monitoring, 77.9% of all pediatric ICUs reported having devices for IBP monitoring available for every pediatric ICU bed in their unit; only 57.1% reported actually using IBP monitoring in every patient diagnosed with septic shock.

Lactate trending had an overall good adherence, as 87% of all pediatric ICUs reported performing routine measurements for lactate trend in patients with septic shock. As for central venous saturation, 19.5% of all pediatric ICUs do not measure it routinely, and 42.8% perform intermittent measurements only in selected patients.

Availability of other methods of invasive hemodynamic monitoring was similar in both groups, as 75% of private/philanthropic and 78.8% of public (SUS) units reported not having any other method available.

All responses regarding hemodynamic monitoring practices are detailed in table 4.

**Table 4:** Monitoring practices

		All pediatric ICUs n (%)	Private/philanthropic n (%)	Public (SUS) n (%)	p value
Availability of a specific protocol in the unit to guide resuscitation and management of patients with septic shock					
	Yes, and all professionals follow the unit's protocol	38 (49.4)	27 (61.3)	11 (33.3)	0.015
	Yes, there is a protocol, but there is no management and/or training of staff to apply it	22 (28.6)	9 (20.4)	13 (39.4)	0.07
	No protocol available	17 (22)	8 (18.3)	9 (27.2)	0.34
In accordance with the current protocol in the unit, with the practical reality of your ICU, based on a personal opinion					
	The protocol is fit for our practical reality	53 (88.3)	34 (94.4)	19 (79.2)	0.06
	Protocol is NOT fit for our practical reality	7 (11.7)	2 (5.6)	5 (20.8)	0.1
Availability of devices for invasive blood pressure monitoring					
	Available for all pediatric ICU beds	60 (77.9)	35 (79.5)	25 (75.7)	0.69
	Some monitors are available in the unit	17 (22.1)	9 (20.5)	8 (24.3)	0.69
	Not available	0	0	0	
Proportion of invasive blood pressure monitoring devices used in patients diagnosed with septic shock					
	Used in all patients	44 (57.1)	25 (56.8)	19 (57.5)	0.94
	Used only in patients with refractory shock	17 (22.1)	11 (25)	6 (18.2)	0.47
	Used only in patients who are on invasive mechanical ventilation	12 (15.6)	7 (15.5)	5 (15.2)	0.92
	Not used	4 (5.2)	1 (2.7)	3 (9.1)	0.18
Routine practice of central venous saturation measures in patients with septic shock					
	Measured continuously for all patients.	3 (3.9)	2 (4.5)	1 (3)	0.73
	Measured intermittently for all patients	26 (33.8)	17 (38.6)	9 (27.3)	0.29
	Measured intermittently for some selected patients	33 (42.8)	20 (45.5)	13 (39.4)	0.59
	Not routinely measured	15 (19.5)	5 (11.4)	310 (0.3)	0.038
Routine practice of lactate concentration measurement in patients with septic shock					
	Lactate measurement is routine, and more than one measurement is taken for comparison (lactate trend)	67 (87)	36 (81.8)	31 (94)	0.11
	Only isolated measurements are taken	8 (10.4)	6 (13.7)	2 (6)	0.28
	Routine measurement is not performed	2 (2.6)	2 (4.5)	0	0.21
Availability of other methods of invasive hemodynamic monitoring of cardiac output (eg : PiCCO, Swan-Ganz, LIDCO, EV 1000)					
	Yes	18 (23.4)	11 (25)	7 (21.2)	0.69
	No	59 (76.6)	33 (75)	26 (78.8)	0.69

ICU - intensive care unit; SUS - *Sistema Único de Saúde*; PiCOO - Pulse index Contour Continuous Cardiac Output; LIDCO - Lithium Dilution Cardiac Output.

### Fluid strategy assessment and vasoactive initiation practices

Across all pediatric ICUs, fluid strategies favored the use only in selected patients rather than the routine use of hemodynamic monitoring parameters.

Specifically, 66.7% of public (SUS) units and 52.3% of private/philanthropic units reported using inferior vena cava (IVC)-based measurements (diameter and/or distensibility/collapsibility) to guide fluid resuscitation only in selected patients, but only 9.1% of public (SUS) units and 18.2% of private/philanthropic units reported using those measurements in all patients.

Considering responses from all pediatric ICUs, regardless of administration type, 40.2% reported not testing for fluid responsiveness before a fluid bolus.

Only 22.1% of total pediatric ICUs reported initiating vasoactive agents, regardless of fluid volume, and 70.1% reported mostly relying on clinical evaluation rather than hemodynamic monitoring for initiation and titration of vasoactive agents.

Detailed answers are presented in tables 5 and 6.

**Table 5:** Volume responsiveness assessment practices

		All pediatric ICUs n (%)	Private/philanthropic n (%)	Public (SUS) n (%)	p value
Proportion of professionals trained in bedside echocardiogram who use IVC diameter measurement and/or the qualitative assessment of IVC distensibility/collapsibility used as a parameter to guide volume resuscitation					
	In all patients	11 (14.3)	8 (18.2)	3 (9.1)	0.26
	Only in selected patients.	45 (58.4)	23 (52.3)	22 (66.7)	0.22
	Do not use	21 (27.3)	13 (29.5)	8 (24.2)	0.6
Proportion of pediatric ICUs that test fluid responsiveness assessed before volume expansion					
	In all patients	5 (6.5)	2 (4.5)	3 (9.1)	0.42
	Only in selected patients.	41 (53.3)	23 (52.3)	18 (54.5)	0.84
	Do not routinely test volume responsiveness	31 (40.2)	19 (43.2)	12 (36.3)	0.54
Preferred method to test fluid responsiveness					
	Through passive elevation of lower limbs and variation of echocardiogram measurements	3 (6.6)	2 (8)	1 (4.8)	0.73
	Through fluid challenge and variation of echocardiogram measurements	28 (60.8)	14 (56)	14 (66.6)	0.34
	Through fluid challenge and variation of clinical parameters	13 (28.2)	8 (8)	5 (23.8)	0.72
	Another method	2 (4.4)	1 (4)	1 (4.8)	0.83

ICU - intensive care unit; SUS - *Sistema Único de Saúde*; IVC - inferior vena cava.

**Table 6:** Fluid therapy practices and vasoactive agents

		All pediatric ICUs n (%)	Private/ philanthropic n (%)	Public (SUS) n (%)	p value
Initial amount of fluids used before starting vasoactive agents					
	20mL/kg	26 (33.7)	13 (29.5)	13 (39.4)	0.36
	30mL/kg	8 (10.4)	4 (9.1)	4 (12.2)	0.66
	40mL/kg	26 (33.7)	18 (40.9)	8 (24.2)	0.12
	Initiates regardless of initial fluids	17 (22.1)	9 (20.5)	8 (24.2)	0.69
Method of choice for starting vasoactive agents					
	Only clinical criteria, based on protocol	58 (75.3)	37 (84.1)	21 (63.6)	0.039
	Based on other forms of hemodynamic monitoring	5 (6.5)	4 (9.1)	1 (3.1)	0.28
	Based on echocardiography	14 (18.2)	3 (6.8)	11 (33.3)	0.03
Most frequently used criteria for initiation and titration of vasoactive agents					
	Clinical evaluation	54 (70.1)	30 (68.2)	24 (72.7)	0.66
	Hemodynamic monitoring	23 (29.9)	14 (31.8)	9 (27.3)	0.66

ICU - intensive care unit; SUS - *Sistema Único de Saúde*.

**Figure 4:**
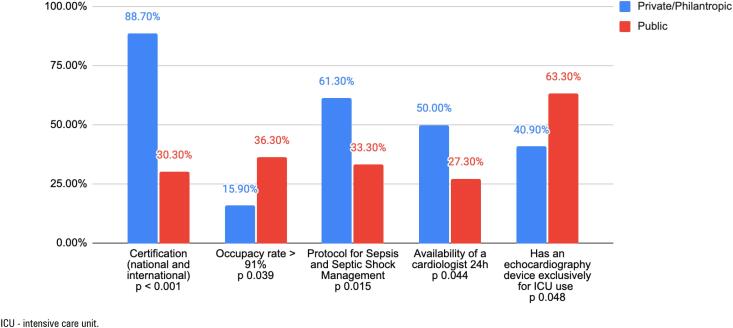
Comparison between private/philanthropic and public (Unified Health System) hospitals for significant variables. The bars represent percentages, and the p-values indicate statistically significant differences (p < 0.05).

Figure 4 shows some visual representation of statistically significant differences in responses between private/philanthropic and public (SUS) hospitals.

### Exploratory association analysis

In bivariate models, units with a non-cardiac clinical+surgical profile had higher odds of not testing for fluid responsiveness before a bolus (OR 2.50, 95%CI 1.10 - 6.40; p = 0.048). Public hospitals had higher odds of not measuring ScvO_2_ in septic shock (OR 3.40, 95%CI 1.03 - 11.10; p = 0.040), as did the non-cardiac profile (OR 3.80, 95%CI 1.09 - 13.20; p = 0.030). No covariate predicted performing serial lactate monitoring or the availability of other advanced hemodynamic methods. Public hospitals were less likely to have a protocol followed by all professionals (OR 0.85, 95%CI 0.013 - 0.85; p = 0.020), whereas accredited hospitals were more likely to have such a protocol (OR 4.14, 95%CI 1.52 - 11.20; p = 0.005). No covariate predicted initiation/titration, mostly guided by hemodynamic monitoring.

In multivariable models, only the outcome "choice of initial vasoactive agent based on echocardiography" retained independent associations: public hospitals (OR 6.30, 95%CI 1.30 - 30.30; p = 0.020) and units where all professionals are specialists (OR 4.46, 95%CI 1.10 - 17.90; p = 0.030). For the other outcomes, no covariate remained an independent predictor.

## DISCUSSION

The results demonstrate a significant discrepancy between clinical practices observed in Brazilian pediatric ICUs and international guidelines, such as the SSC 2020.^(1)^ Although hemodynamic monitoring is widely recognized as important in the management of sepsis and septic shock, our survey highlights that most pediatric ICUs still rely primarily on clinical assessment for initiation and titration of vasoactive agents (70.1%) rather than on hemodynamic monitoring (29.9%).

After the initial fluid bolus, current guidelines recommend interpreting clinical findings with caution and supplementing them with hemodynamic monitoring to categorize shock and select vasoactive agents. They also advise against distinguishing "warm" from "cold" shock based solely on clinical signs, given the dissociation between bedside examination and measured hemodynamics, and the poor correlation between clinical assessment and cardiac index/systemic vascular resistance.^(1,23,24)^ Thus, protocols suggest relying on parameters such as pulse pressure variation, mean arterial pressure (MAP), and diastolic arterial pressure rather than skin temperature, capillary refill time, and pulse quality to classify shock type and decide which vasoactive agent to start.^(1,13,16,24,25)^

Another recommendation is early initiation of vasoactive agents, which may lead to benefits such as improved urine output, increased lactate clearance, and a less positive fluid balance.^(26)^ However, 40.2% of all pediatric ICUs reported not testing fluid responsiveness before a bolus, and only 22% of units responded that they initiate vasoactive agents regardless of the initial fluids, with 33.7% responding that they initiate vasoactive agents after 20mL/kg of fluids, and the other 33.7% only after 40mL/kg of fluids. This delay in starting vasopressors may result in the administration of up to 50mL/kg of extra fluids in some patients when attempting to restore blood pressure and adequate blood flow through fluid replacement alone.^(27,28)^

It is known that, in children with sepsis, fluid boluses can have paradoxical effects of vasodilation, with subsequent worsening of arterial hypotension, promoting damage to the glycocalyx, contributing to greater capillary leak and myocardial dysfunction.^(29-31)^ In addition, fluid overload (> 10%) is an independent predictor of mortality, and patients who require mechanical ventilation and/or vasoactive drugs represent the portion of patients with the highest risk of mortality.^(32,33)^ Considering this knowledge and results obtained in the FEAST trial,^(34)^ the 2020 SSC guidelines^(1)^ already gave recommendations for fluid administration based on pediatric ICU availability, meaning a more conservative approach should be followed in low-resource settings where there is not intensive care readily available, with maintenance fluids instead of fluid boluses being given to non-hypotensive patients and cautious titration of fluid boluses to clinical markers of cardiac output in hypotensive patients.^(1,34)^

The evaluation of hemodynamic status in sepsis must be dynamic rather than static. Once hemodynamic stability is achieved, de-escalation strategies can be employed to remove excess fluid, which can be monitored by serum lactate and capillary refill time to help determine microcirculatory perfusion.^(35-37)^ The combination of these actions - rational fluid administration and timely removal of accumulated fluid - has the potential to actually improve outcomes in children with sepsis.^(36)^

Beyond classification, the SSC 2020^(1)^ also emphasizes the importance of constantly reassessing the patient response to each intervention performed, that is, after administration of fluids as well as initiation of vasopressor or inotropic agents, and that physical examination be complemented by invasive blood pressure monitoring and bedside echocardiography in order to take a more informed decision.^(1,13,16,27,38)^

However, despite near-universal echocardiography availability (94.8% reporting pediatric ICU-dedicated or hospital-shared devices), only 14.3% of pediatric ICUs reported routine POCUS to guide fluid resuscitation, and just 6.5% tested fluid responsiveness in all patients. This availability-use gap points to implementation factors rather than equipment scarcity as the main bottleneck. We hypothesize that this selective use may stem from a perception that clinical examination suffices in many cases, that is, there might be a lack of perceived importance of assessing fluid status in every patient. Such practice may expose patients to avoidable fluid loading and delayed vasopressor initiation when fluid status is not objectively determined before resuscitation.

There is strong evidence that, in the absence of hemodynamic monitoring, providers have limited ability to determine cardiac output and patients’ volume status.^(11)^ The use of POCUS is widely recommended in the management of septic shock,^(1,39)^ and it is known that diagnostic accuracy can be significantly improved when echocardiographic assessment is added to physical examination.^(40)^ This method is often used in daily clinical practice in high-income countries. Recent clinical trials suggest that early use of bedside echocardiography by a trained rapid response team can increase the number of correct diagnoses, reduce time to treatment initiation, and even improve patient survival when used appropriately at the onset of respiratory or circulatory failure.^(11,41)^

Our findings point to implementation gaps at the bedside. Brazilian ICU data and pediatric ICU-focused reviews report that suboptimal adherence to POCUS reflects execution barriers such as adequate training/competency and equipment availability, emphasizing the need for standardized training and credentialing.^(42)^

Brazil is a continental country with substantial regional and structural differences, and there are two types of healthcare systems: a unified healthcare system, which goes by the acronym SUS, which offers universal public healthcare assistance, and private care, which theoretically has more resources but may also have relevant structural deficits.^(43)^

We compared both types of institutions in order to clarify if those structural differences could be the reason for implementation barriers and found heterogeneous resource configurations across hospital types. More public (SUS) units (63.3%) reported having an echocardiography device exclusive for pediatric ICU use, while there is a greater number of hospitals accredited by certifying organizations among private/philanthropic hospitals (88.7%), in addition to those units having a 24-hour cardiology service available (50%) and also 61.3% reported having an institutional protocol for managing patients with sepsis and septic shock, for which 94.4% believe is in accordance with the practical reality of their pediatric ICU.

Most of the units that responded to this study (76.6%) reported not having any other methods for invasive cardiac output monitoring available, and several care processes, such as fluid resuscitation practices, assessment of fluid responsiveness, invasive blood pressure monitoring, and initiation and titration of vasoactive agents, were similar in public (SUS) and private/philanthropic units.

There are more similarities within the groups as there was no statistically significant difference between public and private/philanthropic hospitals regarding the number of specialists working in the units, with less than 50% having teams formed only by pediatric ICU specialists, nor regarding the number of trained professionals to perform POCUS, both groups having less than 40% of units with a trained professional in every shift, even in private care, indicating that the lack of specialists is one of the main reasons for an underutilization of resources. From this, it is understood that experienced professionals trained in the identification and management of sepsis are as important as the availability of resources for implementing a quality service with evidence-based practices and for reducing mortality from treatable conditions.^(29,44)^

Following institutional guidelines for sepsis management is one of the most effective ways to reduce mortality rates, achieved by standardizing key aspects of care, optimizing cardiovascular assessment methods, minimizing errors in assistance, and preventing avoidable complications.^(45,46)^ In this study, 23% of all units reported not having a protocol for sepsis and septic shock management in their pediatric ICUs or institution, in addition to 28% who reported having an institutional protocol, but without proper management or team training to actually apply it.

In 2017, a review of the 2007 American College of Critical Care Medicine (ACCM) recommendations was conducted, noting significant variation in the algorithms used across institutions, which depended on the complexity of hospitals and the allocation of resources in each location. Although it is reasonable for each institution to have its own algorithms, it is understood that some key points are necessary for the recognition, management, and stabilization of patients with sepsis and septic shock.^(15)^ Recommendations such as the use of serial bedside echocardiography,^(1,13,40,41)^ insertion of arterial catheter for pulses counter analysis and to help achieve MAP targets,^(1,23)^ measurements of central venous saturation,^(1)^ use of Pulse Contour Cardiac Output (PiCCO), Pulmonary Artery Catheter and other forms of cardiac output monitoring,^(15,45)^ are already well established as forms of hemodynamic monitoring that contribute to more accurate assessment of patient physiology and better outcomes in septic patients.^(1,15,47)^

The 2021 Latin American Consensus on Sepsis, in turn, presented slightly more flexible recommendations regarding hemodynamic monitoring methods, taking into account the particularities and limitations of specific regions.^(6)^ Therefore, it would be feasible to create institutional protocols based on the resources available in each location, ensuring standardized care within each unit, based on key recognition points and specific treatment goals that need to be achieved, regardless of the hemodynamic monitoring methods used. Studies conducted in low- and middle-income countries, along with actions such as implementing training programs and adapting international protocols to local realities, can contribute to improving clinical outcomes in pediatric patients with sepsis and septic shock in Brazil.

This study has limitations related to its method, which uses self-reported, non-audited data; it is important to keep in mind that reported adherence to guidelines/protocols may not reflect real-world practice. Our questionnaire distinguished availability from routine use, but social desirability and recall can still bias responses toward perceived best practice. This consideration tempers the interpretation of seemingly high protocol availability and may help explain the persistent reliance on clinical signs for fluid boluses and the initiation of vasoactive agents.

The sampling frame is subjected to a convenience bias as it comprised the BRnet-PIC network with participation of 77 out of the 107 (72%) registered pediatric ICUs in the network, with 63.7% of the answers concentrated in the Southeast region.

For reference, Brazil has 209 pediatric ICUs registered in the EpiMed Database®,^(22)^ taking that number into account, our study had a participation of 36.8% of the total number, that accounting for 33 out of 84 public (SUS) pediatric ICUs (39.2%) from the North, Northeast and Central-West regions of the country, which are the regions with the greatest scarcity of resources.^(48)^ If we take it a step further and consider Brazil has 425 pediatric ICUs registered at the 2025 DATASUS report^(21)^ our study counted with a participation of only 18% of the total pediatric ICUs in the country and an even lower number of responses, only 21 out of 161 existing pediatric ICUs^(21)^ in the most resource-limited regions in Brazil.^(48)^

In other words, relative to national frames, our sample showed regional imbalances, with an over-representation of the Southeast and under-representation of the North, Northeast, and Center-West regions, which limits our ability to extrapolate results to those regions.

Given voluntary participation and a limited sample within a Brazilian pediatric ICU research network, our findings may represent a best-case scenario of current practices in Brazil, as units with protocols or advanced technologies may have been more likely to respond, potentially overestimating adherence and resource availability. Likewise, the EpiMed Database®/AMIB census^(46)^ cited for context is itself a convenience sample of participating units, not a census of national capacity.

Therefore, even with results that indicate low adherence to monitoring protocols, convenience sampling and a response rate below 50% relative to the estimated total of units may limit generalizability and indicate that we may still have overestimated relevant data, such as the percentage of units that have a whole team of specialists (42.3%) or at least the majority of specialists (> 50% of the team) working in the unit (32.1%), which means those numbers could be even lower, considering that most units that responded were in the southeast region, one of the most developed regions of Brazil.^(43,48)^ A smaller number of specialists working in pediatric ICUs may mean that fluid therapy and hemodynamic monitoring practices are even further away from those recommended by guidelines.

That being said, our sampling frame was the BRnet-PIC network, not the entire country, so we do not claim national representativeness. Results should therefore be interpreted as descriptive of participating network pediatric ICUs rather than fully representative of all Brazilian pediatric ICUs.

Bridging the gap between recommendations and bedside practice will require pragmatic implementation studies and multicenter quality-improvement initiatives that combine standardized POCUS/hemodynamic training, adapted guidelines for limited-resource settings and practice patterns, and prioritizing underrepresented regions and measuring both process and patient-centered outcomes.

## CONCLUSIONS AND PERSPECTIVES

This study portrays a significant discrepancy between clinical practices in Brazilian pediatric intensive care units and international recommendations for the management of pediatric sepsis and septic shock, highlighting the overreliance on clinical assessment rather than hemodynamic monitoring and the underutilization of advanced tools such as point-of-are cardiac ultrasound, which may compromise the optimization of fluid resuscitation and timely initiation of vasoactive agents. A comparison between public and private/philanthropic hospitals highlighted heterogeneous resource configurations but broadly similar processes of care, suggesting that gaps in implementation and workforce training, rather than equipment alone, are the predominant barriers. To translate these gaps into measurable improvement, pragmatic implementation studies and multicenter quality-improvement initiatives are warranted to tailor recommendations for resource-limited settings, evaluate impact, and sustain adherence to guideline-concordant care.

## Data Availability

Data is available on demand from referees.

## References

[1] Weiss SL, Peters MJ, Alhazzani W, Agus MS, Flori HR, Inwald DP, et al. Surviving sepsis campaign international guidelines for the management of septic shock and sepsis-associated organ dysfunction in children. Intensive Care Med. 2020;46(Suppl 1):10-67. 10.1007/s00134-019-05878-6 PMC7095013 32030529

[2] de Souza DC, Gonçalves Martin J, Soares Lanziotti V, de Oliveira CF, Tonial C, de Carvalho WB, et al.; SPREAD PED Investigators and the Instituto Latino Americano de Sepsis Network. The epidemiology of sepsis in paediatric intensive care units in Brazil (the Sepsis PREvalence Assessment Database in Pediatric population, SPREAD PED): an observational study. Lancet Child Adolesc Health. 2021;5(12):873-81. 10.1016/S2352-4642(21)00286-8 34756191

[3] Sanchez-Pinto LN, Bennett TD, DeWitt PE, Russell S, Rebull MN, Martin B, et al. Development and Validation of the Phoenix Criteria for Pediatric Sepsis and Septic Shock. JAMA. 2024;331(9):675-86. 10.1001/jama.2024.0196 PMC10900964 38245897

[4] Schlapbach LJ, Watson RS, Sorce LR, Argent AC, Menon K, Hall MW, et al.; Society of Critical Care Medicine Pediatric Sepsis Definition Task Force. International Consensus Criteria for Pediatric Sepsis and Septic Shock. JAMA. 2024;331(8):665-74.

[5] Rodrigues LD, Rodrigues LD, Costa LC, Fontoura GM, Maciel MC. Trend in infant mortality rate caused by sepsis in Brazil from 2009 to 2018. Rev Inst Med Trop São Paulo. 2021;63:e26. 10.1590/S1678-9946202163026 PMC8046506 33852709

[6] Fernández-Sarmiento J, De Souza DC, Martinez A, Nieto V, López-Herce J, Soares Lanziotti V, et al. Latin American Consensus on the Management of Sepsis in Children: Sociedad Latinoamericana de Cuidados Intensivos Pediátricos [Latin American Pediatric Intensive Care Society] (SLACIP) Task Force: Executive Summary. J Intensive Care Med. 2022;37(6):753-63. 10.1177/08850666211054444 34812664

[7] Khilnani P, Singhi S, Lodha R, Santhanam I, Sachdev A, Chugh K, et al. Pediatric Sepsis Guidelines: summary for resource-limited countries. Indian J Crit Care Med. 2010;14(1):41-52. 10.4103/0972-5229.63029 PMC2888329 20606908

[8] Watson RS, Carrol ED, Carter MJ, Kissoon N, Ranjit S, Schlapbach LJ. The burden and contemporary epidemiology of sepsis in children. Lancet Child Adolesc Health. 2024;8(9):670-81. 10.1016/S2352-4642(24)00140-8 39142741

[9] Abraham E. New Definitions for sepsis and septic shock: continuing evolution but with much still to be done. JAMA. 2016;315(8):757-9. 10.1001/jama.2016.0290 26903333

[10] Menon K, Schlapbach LJ, Akech S, Argent A, Biban P, Carrol ED, et al.; Pediatric Sepsis Definition Taskforce of the Society of Critical Care Medicine. Criteria for Pediatric Sepsis-A Systematic Review and Meta-Analysis by the Pediatric Sepsis Definition Taskforce. Crit Care Med. 2022;50(1):21-36. 10.1097/CCM.0000000000005294 PMC8670345 34612847

[11] Ip PY, Periasamy U, Staffa SJ, Zurakowski D, Kantor DB. Management changes after echocardiography are associated with improved outcomes in critically ill children. Pediatr Crit Care Med. 2024;25(8):689-98. 10.1097/PCC.0000000000003513 38591948

[12] 12. Razavi A, Newth CJ, Khemani RG, Beltramo F, Ross PA. Cardiac output and systemic vascular resistance: clinical assessment compared with a noninvasive objective measurement in children with shock. J Crit Care. 2017;39:6 - 10. 10.1016/j.jcrc.2016.12.018 28088009

[13] Ranjit S, Aram G, Kissoon N, Ali MK, Natraj R, Shresti S, et al. Multimodal monitoring for hemodynamic categorization and management of pediatric septic shock. Pediatr Crit Care Med. 2014;15(1):e17-26. 10.1097/PCC.0b013e3182a5589c 24196006

[14] Weiss SL, Balamuth F, Hensley J, Fitzgerald JC, Bush J, Nadkarni VM, et al. The epidemiology of hospital death following pediatric severe sepsis: when, why, and how children with sepsis die. Pediatr Crit Care Med. 2017;18(9):823-30. 10.1097/PCC.0000000000001222 PMC5581233 28549024

[15] Davis AL, Carcillo JA, Aneja RK, Deymann AJ, Lin JC, Nguyen TC, et al. American College of Critical Care Medicine Clinical Practice Parameters for Hemodynamic Support of Pediatric and Neonatal Septic Shock. Crit Care Med. 2017;45(6):1061-93. 10.1097/CCM.0000000000002425 28509730

[16] Bigoni A, Cunha AR, Antunes JL. Provision of health resources in the intermediate regions of Brazil, 2018. Cad Saude Colet. 2023;31(4):e31040468.

[17] Vincent JL, Singer M, Einav S, Moreno R, Wendon J, Teboul JL, et al. Equilibrating SSC guidelines with individualized care. Crit Care. 2021;25(1):397. 10.1186/s13054-021-03813-0 PMC8596086 34789298

[18] Carter MJ, Carrol ED, Ranjit S, Mozun R, Kissoon N, Watson RS, et al. Susceptibility to childhood sepsis, contemporary management, and future directions. Lancet Child Adolesc Health. 2024;8(9):682-94. 10.1016/S2352-4642(24)00141-X 39142742

[19] de Souza DC, Paul R, Mozun R, Sankar J, Jabornisky R, Lim E, et al. Quality improvement programmes in paediatric sepsis from a global perspective. Lancet Child Adolesc Health. 2024;8(9):695-706. 10.1016/S2352-4642(24)00142-1 39142743

[20] Vazzoler-Mendonça A, Rondini CA, Costa-Lobo C. Procedimento de avaliação de instrumentos por comitê de juízes especialistas como método de aprimoramento de pesquisa quanti-qualitativa. Rev GESTO-DEBATE. 2023;23(3):47-86.

[21] Brasil. Ministério da Saúde. Sistema Único de Saúde. Cadastro Nacional de Estabelecimentos de Saúde (CNES): TabNet. [citado 2025 Set 1]. Disponível em: https://tabnet.saude.prefeitura.sp.gov.br/cgi/deftohtm3.exe?secretarias/saude/TABNET/cnes/estab.def

[22] Associação de Medicina Intensiva Brasileira (AMIB). Epidemiological profile: National Intensive Care Register in Brazil. São Paulo:AMIB; c2023 [cited 2026 Marc 7]. Available from: https://www.utisbrasileiras.com/en/

[23] Walker SB, Conlon TW, Zhang B, Mensinger JL, Fitzgerald JC, Himebauch AS, et al. Clinical signs to categorize shock and target vasoactive medications in warm versus cold pediatric septic shock. Pediatr Crit Care Med. 2020;21(12):1051-8. 10.1097/PCC.0000000000002481 32740190

[24] Brierley J, Peters MJ. Distinct hemodynamic patterns of septic shock at presentation to pediatric intensive care. Pediatrics. 2008;122(4):752-9. 10.1542/peds.2007-1979 18829798

[25] Ranjit S, Kissoon N, Argent A, Inwald D, Ventura AM, Jaborinsky R, et al. Haemodynamic support for paediatric septic shock: a global perspective. Lancet Child Adolesc Health. 2023;7(8):588-98. 10.1016/S2352-4642(23)00103-7 37354910

[26] Carlos Sanchez E, Pinsky MR, Sinha S, Mishra RC, Lopa AJ, Chatterjee R. Fluids and early vasopressors in the management of septic shock: do we have the right answers yet? J Crit Care Med (Targu Mures). 2023;9(3):138-47. 10.2478/jccm-2023-0022 PMC10425929 37588181

[27] Kim WY, Jun JH, Huh JW, Hong SB, Lim CM, Koh Y. Radial to femoral arterial blood pressure differences in septic shock patients receiving high-dose norepinephrine therapy. Shock. 2013;40(6):527-31. 10.1097/SHK.0000000000000064 24089010

[28] Ranjit S, Natraj R, Kissoon N, Thiagarajan RR, Ramakrishnan B, Monge García MI. Variability in the hemodynamic response to fluid bolus in pediatric septic shock. Pediatr Crit Care Med. 2021;22(8):e448-58. 10.1097/PCC.0000000000002714 33750093

[29] Schultz MJ, Dunser MW, Dondorp AM, Adhikari NK, Iyer S, Kwizera A, et al.; Global Intensive Care Working Group of the European Society of Intensive Care Medicine. Current challenges in the management of sepsis in ICUs in resource-poor settings and suggestions for the future. Intensive Care Med. 2017;43(5):612-24. 10.1007/s00134-017-4750-z 28349179

[30] Ranjit S. Is Rapid bedside echocardiography in septic shock possible? Pediatr Crit Care Med. 2024;25(8):758-61. 10.1097/PCC.0000000000003538 39101800

[31] Rusmawatiningtyas D, Rahmawati A, Makrufardi F, Mardhiah N, Murni IK, Uiterwaal CS, et al. Factors associated with mortality of pediatric sepsis patients at the pediatric intensive care unit in a low-resource setting. BMC Pediatr. 2021;21(1):471. 10.1186/s12887-021-02945-0 PMC8543407 34696763

[32] Fernández-Sarmiento J, Sierra-Zuñiga MF, Salazar González MP, Lucena N, Soares Lanziotti V, Agudelo S. Association between fluid overload and mortality in children with sepsis: a systematic review and meta-analysis. BMJ Paediatr Open. 2023;7(1):e002094. 10.1136/bmjpo-2023-002094 PMC10668252 37989355

[33] Maitland K, George EC, Evans JA, Kiguli S, Olupot-Olupot P, Akech SO, et al.; FEAST trial group. Exploring mechanisms of excess mortality with early fluid resuscitation: insights from the FEAST trial. BMC Med. 2013;11(1):68. 10.1186/1741-7015-11-68 PMC3599745 23496872

[34] Pfortmueller CA, Dabrowski W, Wise R, van Regenmortel N, Malbrain ML. Fluid accumulation syndrome in sepsis and septic shock: pathophysiology, relevance and treatment-a comprehensive review. Ann Intensive Care. 2024;14(1):115. 10.1186/s13613-024-01336-9 PMC11264678 39033219

[35] Fernández-Sarmiento J, Sushitra Ranjit L. Nelson Sanchez-Pinto, Nadkarni VM, Jabornisky R, Kissoon N. The Resuscitation, Equilibrium and De-escalation (RED) strategy: a phased, personalized hemodynamic support in children with sepsis. Front Pediatr. 2025;13:1530984. 10.3389/fped.2025.1530984 PMC11813869 39944316

[36] Messmer AS, Dill T, Müller M, Pfortmueller CA. Active fluid de-resuscitation in critically ill patients with septic shock: A systematic review and meta-analysis. Eur J Intern Med. 2023;109:89-96. 10.1016/j.ejim.2023.01.009 36635127

[37] El-Nawawy AA, Abdelmohsen AM, Hassouna HM. Role of echocardiography in reducing shock reversal time in pediatric septic shock: a randomized controlled trial. J Pediatr (Rio J). 2018;94(1):31-9. 10.1016/j.jped.2017.02.005 28873333

[38] Maitland K, Kiguli S, Opoka RO, Engoru C, Olupot-Olupot P, Akech SO, et al.; FEAST Trial Group. Mortality after fluid bolus in African children with severe infection. N Engl J Med. 2011;364(26):2483-95. 10.1056/NEJMoa1101549 21615299

[39] Manno E, Navarra M, Faccio L, Motevallian M, Bertolaccini L, Mfochivè A, et al. Deep impact of ultrasound in the intensive care unit: the "ICU-sound" protocol. Anesthesiology. 2012;117(4):801-9. 10.1097/ALN.0b013e318264c621 22990179

[40] Zieleskiewicz L, Lopez A, Hraiech S, Baumstarck K, Pastene B, Di Bisceglie M, et al. Bedside POCUS during ward emergencies is associated with improved diagnosis and outcome: an observational, prospective, controlled study. Crit Care. 2021;25(1):34. 10.1186/s13054-021-03466-z PMC7825196 33482873

[41] Niamsanit S, Saengthongpitag T, Uppala R, Sitthikarnkha P, Techasatian L, Saengnipanthkul S. Survey of Thai physicians’ practice in pediatric septic shock. Children (Basel). 2024;11(5):597-7. 10.3390/children11050597 PMC11120040 38790592

[42] Pellegrini JA, Cordioli RL, Grumann AC, Ziegelmann PK, Taniguchi LU. Point-of-care ultrasonography in Brazilian intensive care units: a national survey. Ann Intensive Care. 2018;8(1):50. 10.1186/s13613-018-0397-3 PMC5972134 29808412

[43] Ranjit S, Kissoon N. Challenges and Solutions in translating sepsis guidelines into practice in resource-limited settings. Transl Pediatr. 2021;10(10):2646-65. 10.21037/tp-20-310 PMC8578780 34765491

[44] Ranjit S, Kissoon N. Bedside echocardiography is useful in assessing children with fluid and inotrope resistant septic shock. Indian J Crit Care Med. 2013;17(4):224-30. 10.4103/0972-5229.118426 PMC3796901 24133330

[45] Brierley J, Carcillo JA, Choong K, Cornell T, Decaen A, Deymann A, et al. Clinical practice parameters for hemodynamic support of pediatric and neonatal septic shock: 2007 update from the American College of Critical Care Medicine. Crit Care Med. 2009;37(2):666-88. 10.1097/CCM.0b013e31819323c6 PMC4447433 19325359

[46] Medeiros DN, Mafra AC, Carcillo JA, Troster EJ. A Pediatric Sepsis Protocol Reduced Mortality and Dysfunctions in a Brazilian Public Hospital. Front Pediatr. 2021;9:757721. 10.3389/fped.2021.757721 PMC8633899 34869114

[47] Ranjit S, Natraj R. Hemodynamic management strategies in pediatric septic shock: ten concepts for the bedside practitioner. Indian Pediatr. 2024;61(3):265-75. 38217271

[48] Bahia L, Scheffer M. [The Unified Health System (SUS) and the private assistance sector: interpretations and facts]. Saude Debate. 2018;42(Special 3):158-71.

